# TBAJ-876 Displays Bedaquiline-Like Mycobactericidal Potency without Retaining the Parental Drug’s Uncoupler Activity

**DOI:** 10.1128/AAC.01540-19

**Published:** 2020-01-27

**Authors:** Jickky Palmae Sarathy, Priya Ragunathan, Christopher B. Cooper, Anna M. Upton, Gerhard Grüber, Thomas Dick

**Affiliations:** a Department of Medicine, Yong Loo Lin School of Medicine, National University of Singapore, Singapore; b School of Biological Sciences, Nanyang Technological University, Singapore; c Global Alliance for TB Drug Development (TB Alliance), New York, New York, USA; d Department of Microbiology and Immunology, Yong Loo Lin School of Medicine, National University of Singapore, Singapore; e Center for Discovery and Innovation, Hackensack Meridian Health, Nutley, New Jersey, USA

**Keywords:** TBAJ-876, bedaquiline, uncoupler, protonophore, tuberculosis

## Abstract

The diarylquinoline F_1_F_O_-ATP synthase inhibitor bedaquiline (BDQ) displays protonophore activity. Thus, uncoupling electron transport from ATP synthesis appears to be a second mechanism of action of this antimycobacterial drug. Here, we show that the new BDQ analogue TBAJ-876 did not retain the parental drug’s protonophore activity. Comparative time-kill analyses revealed that both compounds exert the same bactericidal activity. These results suggest that the uncoupler activity is not required for the bactericidal activity of diarylquinolines.

## Abstract (precis)

The diarylquinoline F_1_F_O_-ATP synthase inhibitor bedaquiline (BDQ) displays protonophore activity. Thus, uncoupling electron transport from ATP synthesis appears to be a second mechanism of action of this antimycobacterial drug. Here, we show that the new BDQ analogue TBAJ-876 did not retain the parental drug’s protonophore activity. Comparative time-kill analyses revealed that both compounds exert the same bactericidal activity.

## TEXT

Bedaquiline (BDQ; Sirturo) is a diarylquinoline drug (1) used for the treatment of multidrug-resistant tuberculosis (2, 3). The drug functions by inhibiting Mycobacterium tuberculosis F_1_F_O_-ATP synthase through targeting of both the c (1, 4) and the ε (5–7) subunits. Recently, BDQ was uncovered to be an H^+^/K^+^ antiporter (8). Through its protonophore activity, the drug shuttles protons across the lipid bilayer to collapse the transmembrane pH gradient component of the proton motive force. Elimination of the pH gradient disables the link between electron transport and ATP synthesis (8, 9). BDQ’s uncoupler activity appears to be a second mechanism of action of the drug, contributing to the drug’s bactericidal activity against M. tuberculosis (9).

**FIG 1:**
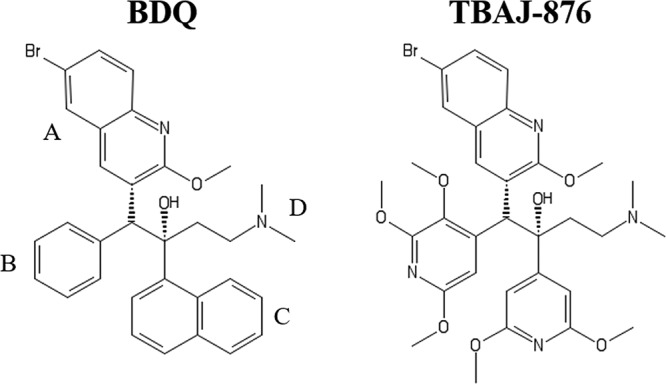
Structures of BDQ and TBAJ-876. TBAJ-876 is described in reference 14. BDQ’s quinoline (A) and dimethylamino (D) groups are retained in TBAJ-876, while its phenyl (B) and naphthalene (C) groups are replaced by 2,3,5-trialkoxypyridin-4-yl and 3,5-dialkoxypyridin-4-yl groups, respectively.

Recent medicinal chemistry campaigns led to the discovery of 3,5-dialkoxypyridine analogues of BDQ with improved pharmacological and toxicological properties (10–14). TBAJ-876 (Fig. 1) is a developmental compound of this series that displays attractive efficacy in a murine model of tuberculosis (14). Biochemical, genetic, and biophysical mechanism-of-action studies confirmed that TBAJ-876 retains the mycobacterial F_1_F_O_-ATP synthase as its target (15).

BDQ translocates protons across membranes in a similar manner as the weakly basic protonophore ellipticine (8, 16). Structure-activity relationship studies of protonophores in the context of mitochondrial toxicity showed that high lipophilicity is critical to their ability to pass through lipid bilayers (17). Since TBAJ-876 is much less lipophilic than BDQ (cLogP of 5.15 versus 7.25) (14), we hypothesized that TBAJ-876 may have lost BDQ’s property of translocating protons.

**FIG 2:**
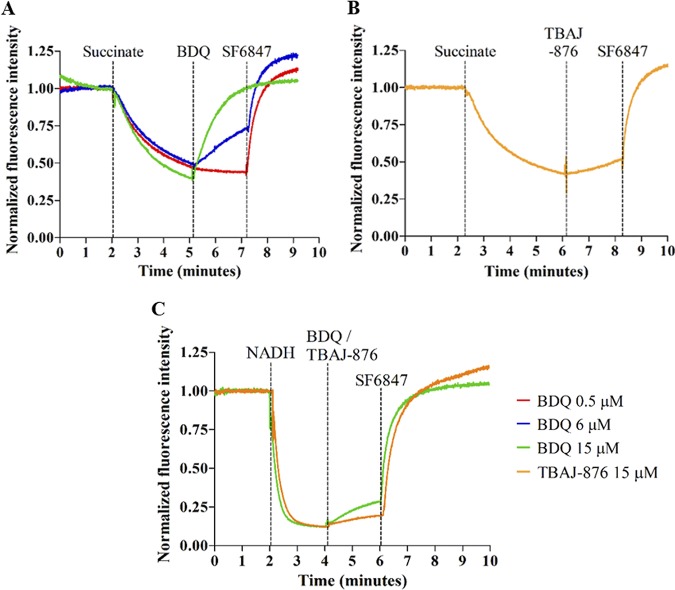
Effects of BDQ and TBAJ-876 on the transmembrane pH gradient of inverted vesicles prepared from M. smegmatis plasma membrane. Shown are the effects of 0.5, 6, and 15 μM BDQ (A and C) or 15 μM TBAJ-876 (B and C) on the quenching of fluorescence of the pH-sensitive fluorophore ACMA. At the beginning of the experiments, 0.5 mM succinate (A and B) or 2 mM NADH (C) was added as an electron donor to the vesicle samples. The inverted vesicles oxidized succinate/NADH and pumped protons to generate the transmembrane pH gradient, visualized as quenching of fluorescence; 1 μM uncoupler SF6847 was added at the end of each experiment as a positive control to collapse the transmembrane pH gradient. The vertical dotted lines indicate the time points at which succinate, NADH, BDQ, TBAJ-876, or SF6847 was added. The experiments were carried out twice independently, showing the same result. Data from a representative replicate are shown. The graphs were generated using GraphPad Prism 5 software. The MIC_90_s for BDQ and TBAJ-876 against M. smegmatis are 100 nM and 6.3 nM, respectively, as described in reference 15.

To determine whether TBAJ-876 displays protonophore activity, we measured the effect of the compound on the transmembrane pH gradient of mycobacterial inverted vesicles using the pH-responsive fluorophore 9-amino-6-chloro-2-methoxyacridine (ACMA) (Sigma-Aldrich, USA) as described previously (9). The vesicles were generated from plasma membrane preparations isolated from cultures of Mycobacterium smegmatis mc^2^ 155 (ATCC 700084) as described previously (18). As published by Hards et al. (9), succinate (Sigma-Aldrich, USA) was used as an electron donor to energize the vesicles. The addition of 0.5 mM succinate resulted in quenching of ACMA fluorescence, thus indicating pH change due to the establishment of the pH gradient across the vesicles’ membrane (Fig. 2). One micromolar of the bona fide protonophore SF6847 (Sigma-Aldrich, USA) was used as a positive control to collapse the pH gradient at the end of each experiment, as detected by the loss of fluorescence quenching (Fig. 2). BDQ (MedChemExpress, USA) caused a dose-dependent reduction of ACMA fluorescence quenching (Fig. 2A). Fifteen micromolar of the drug caused a complete reversal of quenching, thus indicating elimination of the pH gradient (Fig. 2A). These two observations are consistent with previous reports on BDQ’s effect on the transmembrane pH gradient (8, 9). Hence, these results confirm that BDQ displays protonophore activity and thus uncouples electron transport from ATP synthesis. In contrast, 15 μM TBAJ-876 reduced quenching by only 20% and thus had a much weaker effect on the transmembrane pH gradient than BDQ (Fig. 2B). The effect of BDQ and TBAJ-876 on the transmembrane pH gradient was also assessed using NADH as an electron donor to energize the vesicles. The uncoupling effect of 15 μM BDQ was drastically reduced in NADH-energized membrane vesicles (Fig. 2C). This is consistent with a previous report by Hards and Cook who showed that the uncoupling effect of BDQ is influenced by the electron donor used for respiration (19). Consistent with the results from the succinate-energized vesicles, 15 μM TBAJ-876 had a weaker effect than BDQ on the transmembrane pH gradient in NADH-energized vesicles (Fig. 2C). Collectively, the results suggest that TBAJ-876 did not retain BDQ’s pronounced protonophore activity.

**FIG 3:**
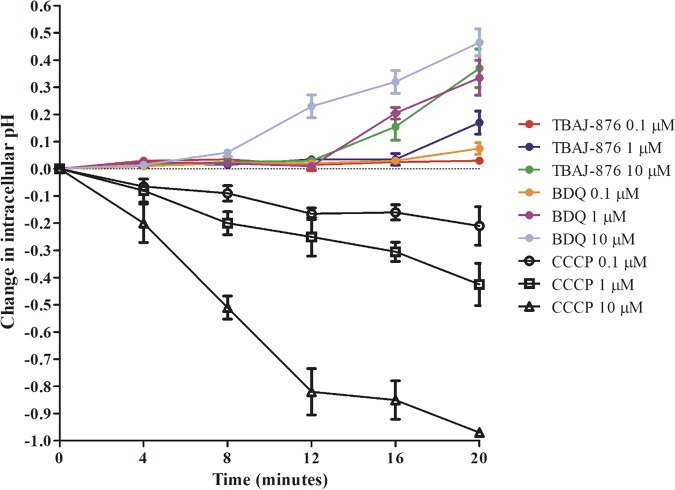
Effect of BDQ and TBAJ-876 on intracellular pH of whole-cell M. bovis BCG. Shown are the effects of 0.1, 1, and 10 μM BDQ or TBAJ-876 on intracellular pH over time in the absence of a pH gradient between the cell interior and exterior. Carbonyl cyanide *m*-chlorophenyl hydrazine (CCCP), a weakly acidic protonophore previously shown to cause intracellular acidification (22), was used as a positive control. The experiment was carried out three times independently, and the results are presented as mean values with standard deviations. The graph was generated using GraphPad Prism 5 software. The MIC_90_s for BDQ and TBAJ-876 against M. bovis BCG are 70 nM and 7.2 nM, respectively, as described in reference 15. The differences in intracellular pH of samples at the last time point (20 min), treated with the same concentrations of TBAJ-876 or BDQ, were analyzed for statistical significance using unpaired Student's *t* test. The difference in the samples treated with 1 μM antibiotics was significant (*P* < 0.05), whereas the differences in the samples treated with 0.1 and 10 μM were not significant.

The weaker protonophore activity of TBAJ-876 may be a result of its less lipophilic character, limiting its ability to diffuse through lipid-rich membranes and thus preventing it from acting as an efficient proton translocator. In a previous study, it was shown that the weakly basic BDQ readily crosses lipid bilayers to accumulate within the cell interior. This was detected by rapid alkalization of the interior of Escherichia coli liposomes by 1 μM BDQ in the absence of a pH gradient between the liposome interior and exterior (8). If TBAJ-876 (also a weak base) is indeed less effective in diffusing through lipid bilayers than BDQ, the compound should be less effective in causing intracellular alkalization. To test this, we treated Mycobacterium bovis bacillus Calmette-Guérin (BCG; ATCC 35734) cultures with 0.1, 1, and 10 μM TBAJ-876 or BDQ and measured the effects on intracellular pH, in the absence of a pH gradient between the cell interior and exterior, using the pH-sensitive fluorophore 5-chloromethylfluorescein diacetate (CMFDA; Invitrogen, USA) as described previously (20). Consistent with the previous E. coli liposome study (8), treatment with 1 μM BDQ caused a rapid intracellular pH increase of 0.35 units within 20 min (Fig. 3). In contrast, 1 μM TBAJ-876 had a weaker effect (0.17 pH unit increase), and treatment with 10 μM was required to cause a 0.35 unit increase of intracellular pH (Fig. 3). These data support the notion that TBAJ-876 is indeed less effective than BDQ in crossing membranes and are consistent with the compound’s weaker protonophore activity observed in the inverted vesicle experiments.

**FIG 4:**
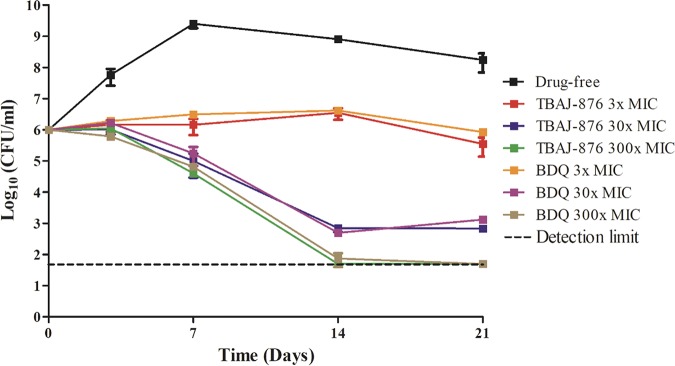
Comparison of the bactericidal activity of BDQ and TBAJ-876 against M. tuberculosis. Time-kill curves of 3-, 30-, and 300-fold the MIC_90_ of TBAJ-876 and BDQ against M. tuberculosis H37Rv are shown. The MIC_90_ values for BDQ and TBAJ-876 are 400 nM and 125 nM, as described in reference 15. The experiment was carried out three times independently, and the results are presented as mean values with standard deviations. The graph was generated using GraphPad Prism 5 software. The minor differences in CFU of samples at the last time point (21 days), treated with the same fold MIC_90_ of TBAJ-876 or BDQ, were analyzed for statistical significance (*P* < 0.05) using unpaired Student's *t* test. No significant differences were found with the exception of the samples treated with 30-fold MIC_90_ of the antibiotics.

The uncoupler activity is proposed to contribute to BDQ’s bactericidal activity (9). Hence, the weaker protonophore activity in TBAJ-876 should reduce the compound’s bactericidal activity. To determine the impact of the weakened protonophore activity on TBAJ-876’s bactericidal activity, we carried out comparative time-kill experiments with M. tuberculosis H37Rv (ATCC 27294) as described previously (21). Cultures were treated over 21 days with 3-, 30-, or 300-fold the MIC_90_ of BDQ or TBAJ-876. Subsequently, bacterial viability was determined by enumeration of CFU. Surprisingly, the kill curves generated by BDQ and TBAJ-876 were indistinguishable, showing that both molecules exert the same bactericidal activity (Fig. 4). This result suggests that the reduced uncoupler activity of TBAJ-876 does not affect the compound’s bactericidal activity.

In conclusion, we show that the 3,5-dialkoxypyridine BDQ analogue TBAJ-876 does not retain the parental drug’s strong uncoupler activity. This is likely due to TBAJ-876’s lower lipophilicity, limiting the compound’s ability to diffuse through lipid bilayers and consequently weakening the compound’s protonophore activity. Unexpectedly, loss of uncoupler activity did not affect the bactericidal activity of TBAJ-876. Our results suggest that uncoupler activity is not critical for members of the diarylquinoline class to exert their antimycobacterial activity.
